# Log Sequence Anomaly Detection Method Based on Contrastive Adversarial Training and Dual Feature Extraction

**DOI:** 10.3390/e24010069

**Published:** 2021-12-30

**Authors:** Qiaozheng Wang, Xiuguo Zhang, Xuejie Wang, Zhiying Cao

**Affiliations:** School of Information Science and Technology, Dalian Maritime University, Dalian 116026, China; wqz@dlmu.edu.cn (Q.W.); wxj@dlmu.edu.cn (X.W.)

**Keywords:** adversarial training, contrastive learning, statistical features, VAE, BERT

## Abstract

The log messages generated in the system reflect the state of the system at all times. The realization of autonomous detection of abnormalities in log messages can help operators find abnormalities in time and provide a basis for analyzing the causes of abnormalities. First, this paper proposes a log sequence anomaly detection method based on contrastive adversarial training and dual feature extraction. This method uses BERT (Bidirectional Encoder Representations from Transformers) and VAE (Variational Auto-Encoder) to extract the semantic features and statistical features of the log sequence, respectively, and the dual features are combined to perform anomaly detection on the log sequence, with a novel contrastive adversarial training method also used to train the model. In addition, this paper introduces the method of obtaining statistical features of log sequence and the method of combining semantic features with statistical features. Furthermore, the specific process of contrastive adversarial training is described. Finally, an experimental comparison is carried out, and the experimental results show that the method in this paper is better than the contrasted log sequence anomaly detection method.

## 1. Introduction

Log messages have become an essential resource for the reliability and continuity of many software systems, especially large-scale distributed systems. It truly reflects the operating status of the software system, and is of great significance to the monitoring, management and troubleshooting of the system. Therefore, log-based anomaly detection has become an important means to ensure system reliability and service quality, and its purpose is to find abnormal behavior of the system [1]. At present, due to the scale and complexity of modern software systems, the volume of logs has reached an unprecedented level. Therefore, for anomaly detection based on log sequences, traditional manual detection methods become impractical. With the development of artificial intelligence technology, some artificial intelligence applications, such as intelligent operation and maintenance [2,3,4,5] and intelligent transportation [6], have become hot research topics. In recent years, some log sequence anomaly detection methods [2,3,4,5] have improved a lot of efficiency compared with manual detection, but they cannot fully extract the information contained in the log text, nor can they fully analyze and utilize the extracted information, which affects the accuracy of anomaly detection. In contrast, the use of deep learning methods can better learn the relationship between logs in the log sequence [7,8,9,10,11,12,13], and then achieve more accurate log anomaly detection, but they are not sufficiently robust in practice. To detect anomalies, almost all existing approaches require the construction of a detection model using the known log events (i.e., the templates of log messages) and log sequences (i.e., series of log events that record specific execution flows) extracted from the training data. They fail to work with previously unseen log events and log sequences. However, our empirical study has found that real-world log data is unstable, meaning that new but similar log events and log sequences often appear [14]. In order to be able to deal with log events that have never been seen before, people have proposed the use of natural language processing to convert the log template into a semantic vector, and then the use of a deep model to learn the deep relationship contained in the semantics [14,15,16,17]. Then, a robust log anomaly detection that can deal with unknown log events is realized, but the consideration of semantic features increases the difficulty of training deep models. Abnormal logs are usually hidden in a large number of normal logs, which affects the model’s semantic learning of abnormal log sequences. Therefore, it is necessary to further standardize the semantic space of positive and negative samples, and the current log anomaly detection only relying on semantic features to deal with robustness problems is not enough, and there is a lack of a training method specifically for robustness. In addition, the current deep learning methods only focus on the log semantic features of the log sequence [18,19,20], and do not utilize the statistical information contained in the log sequence. The core words in the abnormal log usually do not appear in the normal log or appear in the normal log with a low probability, so the statistical features can help anomaly detection proceed more smoothly.

In summary, the current mainstream log anomaly detection methods still have various problems, which are prominent in the following aspects:

(1) Most log sequence anomaly detection methods do not fully consider the robustness issues caused by the update of the log message template, and lack specialized training on the robustness of log sequence anomaly detection.
(2) Most log sequence anomaly detection methods have not conducted more in-depth research and attention on the semantic features generated by normal and abnormal log sequences, that is, the distance relationship between the semantic features of normal and abnormal log sequences in the semantic space is not used to improve the accuracy of anomaly detection.
(3) Most log sequence anomaly detection methods only use the semantic features of the log sequence to perform anomaly detection, and do not combine the information in the statistical characteristics of the log sequence to improve the accuracy of anomaly detection.

In response to the above problems, this paper proposes an anomaly detection method based on contrastive adversarial training and dual feature extraction, and uses the open source real data sets HDFS and BGL to evaluate the proposed method. The main contributions of this paper are as follows:

(1) This paper uses the FGM (Fast Gradient Method) [21] algorithm to perturb the embedding layer of the BERT [22] model to generate the perturbed semantic features, and then narrow the distance between the semantic features generated by the log sequence before and after the embedding layer is perturbed, so that the model can still obtain the correct anomaly detection results even when the original log sequence has some slight changes. This kind of special training for robustness can make the anomaly detection model obtain good robustness.
(2) Contrastive learning [23] is used to reduce the similarity between the semantic features of normal and abnormal log sequences, so that the semantic features generated by normal and abnormal log sequences are farther in the semantic space, and the difference is greater, thereby improving the accuracy of anomaly detection.
(3) This paper counts the times that each word of the log sequence appears in the normal and abnormal label and uses a VAE [24] to extract the statistical results to obtain statistical features [25]. The statistical features and semantic features of the log sequence are then combined to obtain semantic features enhanced by the statistics features to train the model, and the enhanced semantic features will contain more information, thereby improving the accuracy of model anomaly detection.

The rest of this paper is organized as follows. Section 2 introduces the related research of log anomaly detection, Section 3 summarizes the log anomaly detection method proposed in this paper, Section 4 conducts experimental comparison to evaluate the performance of this paper method, and Section 5 summarizes and prospects.

## 2. Related Research

In order to be able to find the abnormalities generated by the log messages in the system in a timely and convenient manner, a log sequence abnormality detection method that can autonomously and accurately detect the abnormality of the log sequence is urgently needed. The current mainstream log sequence anomaly detection methods can be roughly divided into machine learning methods and deep learning methods.

In the machine learning method, Liang et al. used SVM (Support Vector Machines) [5] to detect anomalies in the log sequence, this method constructs a vector based on the number of various log levels in the log sequence or sliding window, and SVM is used to perform supervised training on vectors and label. During anomaly detection, if the detected log sequence is located above the hyperplane, it will be regarded as an anomaly, but this method cannot cope with the update of the log message template, so it lacks robustness. Lou et al. used the IM (Invariant Mining) [2] method to detect anomalies in the log sequence, which uses a log parser to convert unstructured logs into structured logs. Then, structured log messages are further grouped into log message groups according to the relationship between log parameters, and the invariants mined can reveal what the log messages contain inherent linear characteristics. With these invariants to learn, the newly generated log can be judged, and the log containing these invariants is the normal log, otherwise it will be regarded as an abnormality, but this method does not take into account that the log template update will cause the invariants to be updated accordingly, leading to errors in the detection results, so it also lacks robustness. Moreover, this method cannot fully extract the information contained in the log message, which reduces the accuracy of anomaly detection. Xu et al. used PCA (Principal Component Analysis) [3] to detect anomalies in the log sequence. This method constructs the state ratio vector and the message count vector from the extracted information by selecting appropriate variables and grouping related messages. It then uses the unsupervised method of PCA to mine the feature vectors, and mark each feature vector as normal or abnormal, but this method lacks focus only on the text itself, and lacks the analysis and utilization of the semantics of the text, which affects the accuracy of anomaly detection.

In the deep learning method, Du et al. [7] proposed DeepLog, which uses LSTM to learn the normal pattern of log sequences, and uses the trained model to find those log sequences that deviate from the normal trajectory, thereby achieving the purpose of anomaly detection. However, this method does not have a timely response to the log template update method, so it also lacks robustness, and the method lacks the analysis of log sentence-level semantic features, which reduces the accuracy of anomaly detection. Lu et al. [10] proposed a log sequence anomaly detection method using CNN. The CNN-based anomaly detection model used in this method can automatically learn the event relationship in the system log to achieve the purpose of anomaly detection. However, this method uses logkey2vec to perform log template level vector conversion, and the semantic information at the word level is ignored, which affects the accuracy of the detection results. Zhang et al. [14] proposed LogRobust, which uses Bi-LSTM with attention to detect anomalies in log sequences. This method uses natural language processing to convert log templates into semantic features, which can ensure that appropriate log template updates will not cause major changes to the generated semantic features. This, in turn, increases the robustness of anomaly detection to a certain extent, but this method does not specifically train the robustness of anomaly detection, nor does it utilize the statistical features contained in the log sequence, and does not further improve the robustness and accuracy of anomaly detection. In order to clearly distinguish the work of this paper from other studies, this paper compares the above method with the method in this paper, as shown in Table 1.

## 3. Method Overview

A more robust and accurate log sequence anomaly detection method can help operators more accurately find anomalies in the system. In order to improve the robustness and accuracy of log sequence anomaly detection, this paper proposes a log sequence anomaly detection method based on contrastive adversarial training and dual feature extraction. In this section, the method flow is first summarized, and then the various stages of log anomaly detection are introduced, including log parsing, feature extraction, and an overview of the anomaly detection model based on contrastive adversarial training.

### 3.1. Method Flow

**Figure 1:**
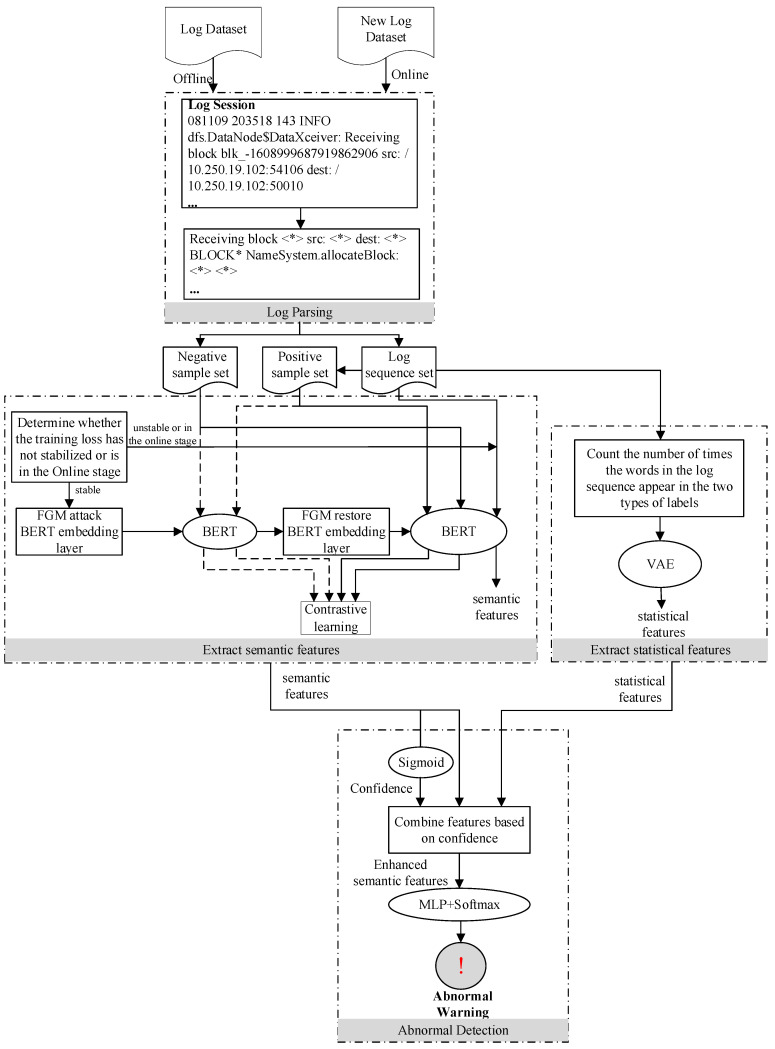
The method flow of CATLog.

This paper proposes a log sequence anomaly detection method CATLog based on contrastive adversarial training and dual feature extraction, that is, the statistical and semantic features of the log sequence are extracted, and the two features are combined to obtain semantic features enhanced by statistical features to train the model. When the loss caused by model training tends to be stable, the FGM algorithm is used to perturb the BERT embedding layer to make the model generate semantic features where, after the perturbation, contrastive learning is used to increase the similarity between the semantic features generated by the normal log sequence before and after the embedding layer of BERT is disturbed. The similarity between the semantic features of all normal and abnormal log sequences is reduced, so as to improve the robustness and accuracy of anomaly detection. The overall process of CATLog is shown in Figure 1.

The steps in the training phase of this method are as follows:

(1) First, the Drain algorithm [26] is used in the log parsing stage to convert unstructured log entries into structured log templates.
(2) Then, the log sequence is obtained according to the session ID or sliding window, and a set of negative sample log sequences is extracted.
(3) Next, in the feature extraction stage, BERT is used to semantically encode the acquired log sequence to obtain semantic features. The number of times each word in the log sequence appears in the normal and abnormal label is counted and entered into the VAE, outputting the hidden variables to obtain statistical features.
(4) The log sequences in the training set and the corresponding labels are used to supervise the training of the anomaly detection model.
(5) When the training loss of the model tends to stabilize, while maintaining the original supervised training task, contrastive adversarial training is used to continue training the model. That is, the FGM algorithm is used to perturb the BERT embedding layer to generate perturbed semantic features. Contrast learning is then used to increase the similarity between the semantic features generated by the normal log sequence when the embedding layer of BERT is not perturbed and perturbed, and the similarity between the semantic features of normal and abnormal log sequences is reduced.

The steps in the detection phase of the method in this paper are as follows:

(1) First, the Drain algorithm is used in the log parsing stage to convert unstructured log entries into structured log templates.
(2) Then, the log sequence is obtained according to the session ID or sliding window.
(3) Next, in the feature extraction stage, BERT is used to semantically encode the acquired log sequence to obtain semantic features. The number of times each word in the log sequence appears in the normal and abnormal label is counted and entered into the VAE, and the hidden variables outputted to obtain statistical features.
(4) The semantic feature is input into the Sigmoid activation function to obtain the confidence, and the statistical feature and the semantic feature are combined according to the confidence to obtain the semantic feature enhanced by the statistical feature.
(5) The enhanced semantic features are input into the trained anomaly detection model and the log sequence is judged on whether it has anomalies according to the output of the model.

### 3.2. Log Analysis

**Figure 2:**
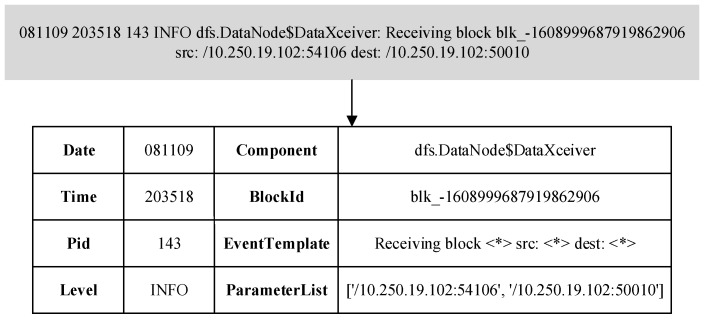
Information contained in log entries.

The log is unstructured and contains any form of text. The purpose of log analysis is to extract a set of event templates so that the original log can be structured. More specifically, the purpose of log analysis is to parse each log message into some specific parameters (variable part) and event templates (constant part) [27]. This paper uses the HDFS public data set as an example to illustrate. The information that can be parsed in a log entry in HDFS is shown in Figure 2.

**Figure 3:**
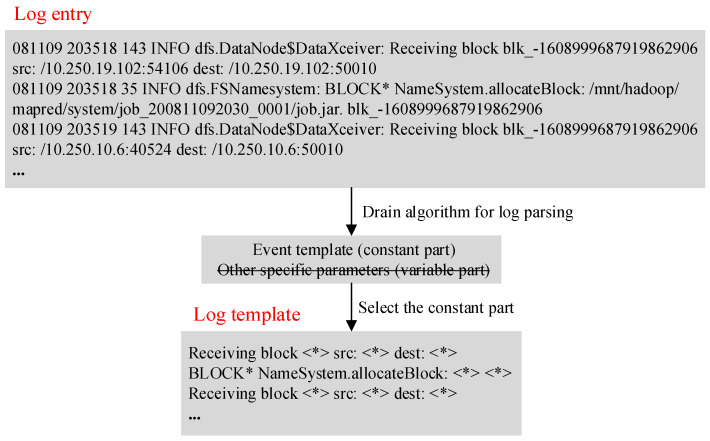
Example of log parsing process.

This paper uses the Drain algorithm to analyze log entries. The algorithm builds a parse tree based on the content of the log entry, and uses the information contained in each layer of the parse tree to determine the log template, thereby converting unstructured log entries into structured log templates. An example of the log parsing process is shown in Figure 3. As can be seen from the figure, the paper uses the Drain algorithm to analyze the unstructured log entries, and obtains the log template (constant part) and other parameters (variable part). In order to conduct in-depth research on the structured log, the variable part is deleted from the parsed information (a line with a cross is used in the figure to indicate deletion) and only the structured log template is output.

### 3.3. Feature Extraction

#### 3.3.1. Semantic Features

**Figure 4:**
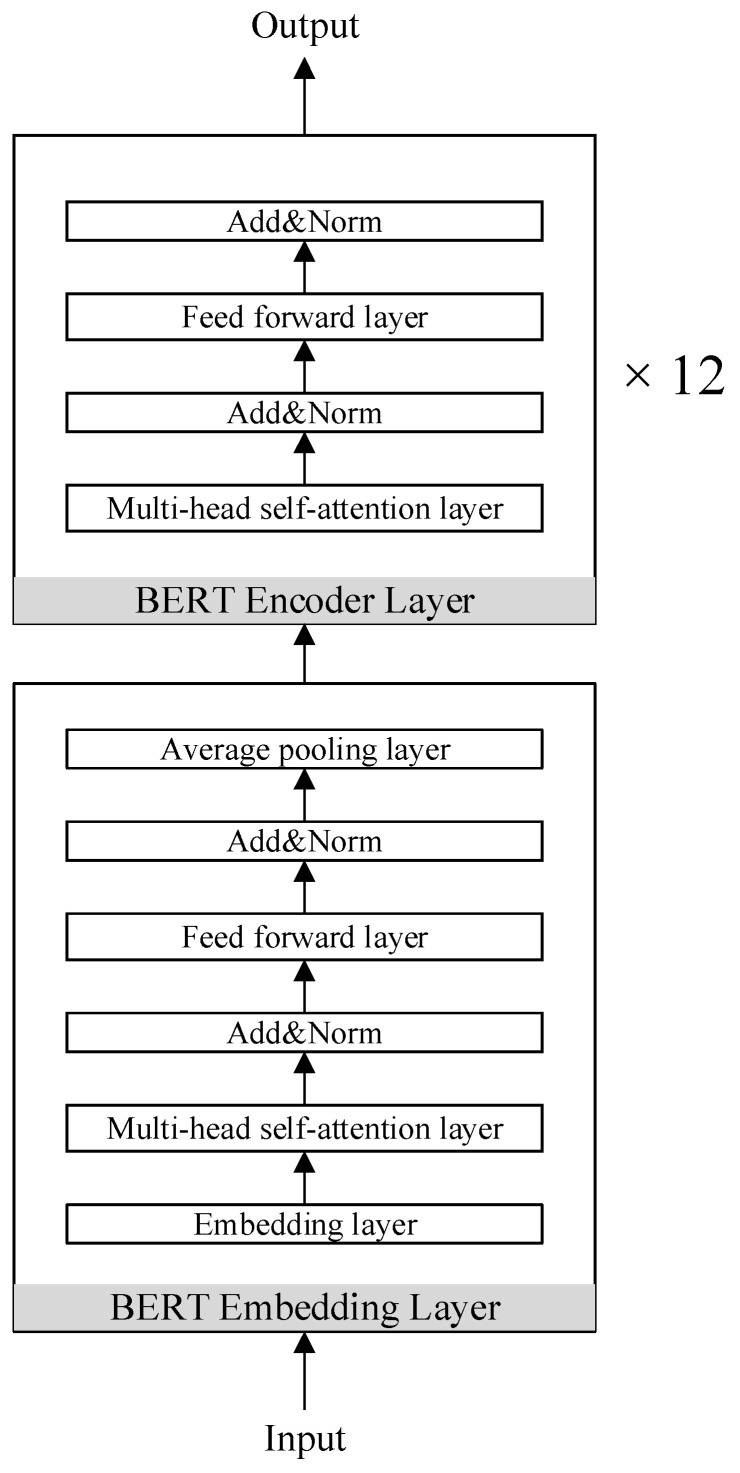
The BERT model with the multi-head self-attention network as the embedding layer.

This paper uses the BERT model to semantically encode the log templates obtained in the log parsing stage to obtain semantic features. The BERT model is generally composed of 12 Encoder layers, and each Encoder layer is completely composed of a multi-head self-attention network and a feedforward neural network. The self-attention network reduces the distance between any two positions in the sequence to a constant, it solves the problem of information loss in the traditional RNN due to the sequential calculation process, and has the ability of parallel calculation, which makes the accuracy of semantic coding higher. Next, this paper will introduce the self-attention network from the perspective of time efficiency. Self-attention includes three steps: similarity calculation, softmax calculation and weighted average. Assuming that n is the length of the sequence and d is the dimension of the word embedding, the similarity calculation can be regarded as the multiplication of two matrices of size (n,d) and (d,n) to obtain a matrix of (n,n), thus the time complexity is $O\left(n^{2}*d\right)$, the time complexity of the softmax calculation is $O\left(n^{2}\right)$, and the weighted average can be regarded as the multiplication of two matrices of size (n,n) and (n,d) to obtain a matrix of (n,d), so the time complexity is $O\left(n^{2}*d\right)$. Therefore, the time complexity of self-attention is $O\left(n^{2}*d\right)$. A multi-head self-attention with m heads is equivalent to dividing the dimension d of the word embedding into m parts and then performing self-attention calculations separately. It is supposed that the dimension of self-attention calculation for each head is d, where a=d/m. On the whole, multi-head self-attention is equivalent to doing m times of (n,a) and (a,n) matrix multiplication, so the time complexity is also $O\left(n^{2}*d\right)$. In order to achieve semantic coding from word to sentence to sequence level, this paper uses a multi-head self-attention network as the embedding layer of the BERT model, so that the log sequence input into the BERT model will convert each word in each log template of the log sequence into a word vector in the embedding layer of the BERT. Then the sentence vector representing the log template is obtained by averaging word vectors and is input into the BERT model. Attention is used to calculate the weight of each vector output by the BERT model. By summing the vectors given different weights, the semantic feature representing the entire log sequence is obtained. The structure of the BERT model with the multi-head self-attention network as the embedding layer is shown in Figure 4.

#### 3.3.2. Statistical Features

In this paper, the statistical results of the number of occurrences of the words in the log sequence in the normal and abnormal label are input into the VAE model, and the hidden variables are output to obtain the statistical features. An autoencoder is a neural network for unsupervised learning that copies inputs to outputs [28], and a variational autoencoder can be defined as being an autoencoder whose training is regularised to avoid overfitting and ensure that the latent space has good properties that enable generative process. In order to facilitate the description, this paper needs to declare some variables used in the process of statistical feature extraction. For a given word, the word statistical vector that counts the number of times it appears in the two types of labels is shown in Formula (1).

(1) $$\zeta^{w}=\left[\zeta_{normal},\zeta_{abnormal}\right]$$

Among them, $\zeta_{normal}$ represents the number of times the word w appears in the normal label, and $\zeta_{abnormal}$ represents the number of times the word w appears in the abnormal label. For a given log sequence $s={\left\{w_{i}\right\}}_{i=1}^{n}$, the log sequence statistical vector that counts the number of times each word in the sequence appears in the two types of labels is shown in Formula (2).

(2) $$\zeta^{s}=\left[\zeta^{w_{1}},\ldots ,\zeta^{w_{n}}\right]$$

Because log sequence statistical vectors are incompatible with semantic features in both dimensions and scales, this paper uses VAE to map discrete statistical vectors to a latent continuous space to obtain a global representation of statistical information, so that it can be combined with semantic features. The optimization objective function of the variational autoencoder is shown in Formula (3), which is composed of a reconstruction term to optimize the encoder and decoder, and a regularization term to regularize the hidden space.

(3) $$\underset{\theta ,\phi }{\max}\left({Ε}_{Z∼q_{\phi }\left(Z|\zeta \right)}\left(\log p_{\theta }\left(\zeta |Z\right)\right)-D_{KL}\left(q_{\phi }\left(Z|\zeta \right)||p_{\theta }\left(Z\right)\right)\right)$$

where ζ is the log sequence statistical vector input to the autoencoder, Z is the hidden variable, $p_{\theta }\left(\zeta |Z\right)$ is the Gaussian distribution of $\zeta^{Z}$ generated from the hidden variable Z, $p_{\theta }\left(Z\right)$ is the prior distribution of selecting the hidden variable, θ is the parameter of the decoder, $q_{\phi }\left(Z|\zeta \right)$ is the Gaussian distribution used to approximate $p_{\theta }\left(Z|\zeta \right)$ in the variational inference process, the mean and covariance are generated by two encoders, and ϕ is the parameter of the encoder. Through unsupervised training of the VAE model, the hidden variable $\zeta^{Z}$ can be obtained, which will become the global representation of the log sequence statistical vector. The training of the VAE model is independent of other parts of the anomaly detection model. The latent variable $\zeta^{Z}$ is generated in the preprocessing stage and is combined with semantic features in the subsequent stage to obtain semantic features enhanced by statistical features. Log anomaly detection can be regarded as a text classification problem to some extent. Generally speaking, the most direct way to improve the effect of text classification is mainly to improve the classification model, or to enhance semantic features through some external or internal information. This paper uses statistical features to enhance semantic features. Statistical features are easier to obtain than other external information and are naturally compatible with the corresponding tasks. The statistical features make the model more certain that the possibility of the input log sequence contains abnormal information, thereby improving the accuracy of anomaly detection.

### 3.4. Anomaly Detection Model Based on Contrastive Adversarial Training

**Figure 5:**
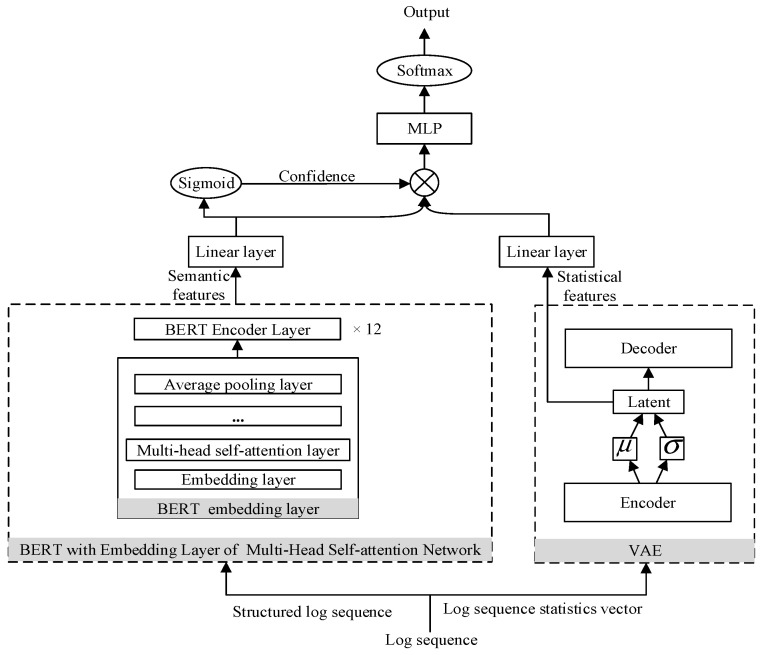
Anomaly detection model.

This paper uses BERT, VAE and MLP to construct an anomaly detection model. The model structure is shown in Figure 5 and the contrastive adversarial training algorithm is shown in Algorithm 1.

**Algorithm 1:** Algorithm for contrastive adversarial training input: Log sequence training set $D={\left\{x_{i},y_{i}\right\}}_{i=1}^{N}$;    Negative sample log sequence $X^{neg}={\left\{x_{j}^{neg}\right\}}_{j=1}^{m}$. output: Trained anomaly detection model.
**1 repeat**
**2**  **for all**$\left\{x_{i},y_{i}\right\}\in D$ **do****3**    Update parameter α with $-\frac{1}{N}\sum_{i=1}^{N}\left(y_{i}\cdot \log\left(f_{\alpha }\left(x_{i}\right)\right)+\left(1-y_{i}\right)\cdot \log\left(1-f_{\alpha }\left(x_{i}\right)\right)\right)$ as the loss function
**4  end**
**5 until** Training loss has stabilized;
**6 repeat**
**7**  **for all**$\left\{x_{i},y_{i}\right\}\in D$**do****8**   Select all normal log sequences from the current batch to form a positive sample log sequence set $X^{\mathrm{pos}}={\left\{x_{k}^{pos}\right\}}_{k=1}^{n}$**9**   Use the FGM algorithm to add a perturbation of $\varepsilon \cdot g/{\left\|g\right\|}_{2}$ to the embedding layer of BERT**10**   Input the set of positive and negative sample log sequences into the BERT that the embedding layer is perturbed to obtain the perturbed semantic vector of the positive and negative sample log sequence ${\left\{v_{k}^{pos,adv}\right\}}_{k=1}^{n}$ and ${\left\{v_{j}^{neg,adv}\right\}}_{j=1}^{m}$**11**   Cancel the disturbance to the BERT embedding layer and input the positive and negative sample log sequences into the BERT again to obtain the semantic vector of the positive and negative sample log sequence ${\left\{v_{k}^{pos}\right\}}_{k=1}^{n}$ and ${\left\{v_{j}^{neg}\right\}}_{j=1}^{m}$**12**   Update parameter α with formula (6) as the optimization function**13**  **end****14 until** Model converges;**15 return** Model with well-trained parameters

The training process of the model is as follows:

(1) The log sequence and the corresponding label in the training set is used to supervise the training of the anomaly detection model.
(2) When the loss generated during training stabilizes, the FGM algorithm is used to perturb the embedding layer of the BERT model.
(3) A negative sample set is constructed by randomly selecting abnormal log sequences from the training set.
(4) The log sequence in the training set and the corresponding label and negative sample set are input into the model.
(5) All normal log sequences in the log sequence of the current batch are selected input to the model to form a positive sample set.
(6) The positive and negative sample set are input into the perturbed BERT model of the embedding layer to obtain the semantic features of the normal and abnormal log sequences after the perturbation, and then cancel the perturbation to the embedding layer.
(7) The positive and negative sample set and the log sequence of the training set are input to the undisturbed BERT model of the embedding layer to obtain the semantic features of the normal and abnormal log sequences and the log sequence of the training set.
(8) While contrastive learning is used to increase the similarity between the semantic features generated by the normal log sequence when the embedding layer of BERT is disturbed and undisturbed, as well as to reduce the semantic features between all normal log sequences and the set of negative sample log sequences, the original supervised training task is continued to train the model. Steps 4 to 8 are repeated until the loss of contrast adversarial training stabilizes.

The detection process of the model is as follows:

(1) The log sequence is input to be detected into the trained anomaly detection model.
(2) The fine-tuned BERT model is used to convert the log sequence to be detected after log parsing into semantic features.
(3) The trained VAE model is used to convert the log sequence statistical vector to be detected into statistical features.
(4) The semantic features and statistical features are input to the fully connected layer so that they become feature vectors of the same dimension.
(5) The semantic features output by the fully connected layer are input into the Sigmoid function to obtain its confidence, and the semantic features and the statistical features are combined according to the confidence to obtain the semantic features enhanced by the statistical features.
(6) The enhanced semantic features are input into the MLP, and the output result of the model is checked to determine whether there is an abnormality in the log sequence.

It can be seen from Figure 5 that after extracting the semantic and statistical features of the log sequence and inputting them to the fully connected layer for unified dimensions, the semantic features output from the fully connected layer will be input to the Sigmoid activation function to obtain confidence. Then, the semantic features and statistical features of the log sequence are combined according to the confidence to obtain the semantic feature enhanced by the statistical feature, where the value of the confidence is obtained by supervised training according to the log sequence and the corresponding label in the training set during the training phase. The calculation method of the combination ratio of statistical features is shown in Formula (4).

(4) $$\mathrm{combine}\left(confidence\right)=\left\{ \begin{array}{l} confidence,\;if\;0.5-\eta \leq confidence\leq 0.5+\eta \\ 0,\;otherwise \end{array}\right.$$

Among them, confidence is the confidence probability of the output of the Sigmoid function, and η is the hyperparameter used to adjust the confidence threshold. The calculation method combining statistical features and semantic features is shown in Formula (5).

(5) $$V^{\mathrm{Enhanced}}=V^{\mathrm{Semantic}}+combine(Sigmoid(V^{\mathrm{Semantic}}))⊙V^{\mathrm{Statistical}}$$

Among them, $V^{\mathrm{Semantic}}$ is a semantic feature, $V^{\mathrm{Statistical}}$ is a statistical feature, $V^{\mathrm{Enhanced}}$ is a semantic feature enhanced by the statistical feature, and ⊙ represents an element-based product.

**Figure 6:**
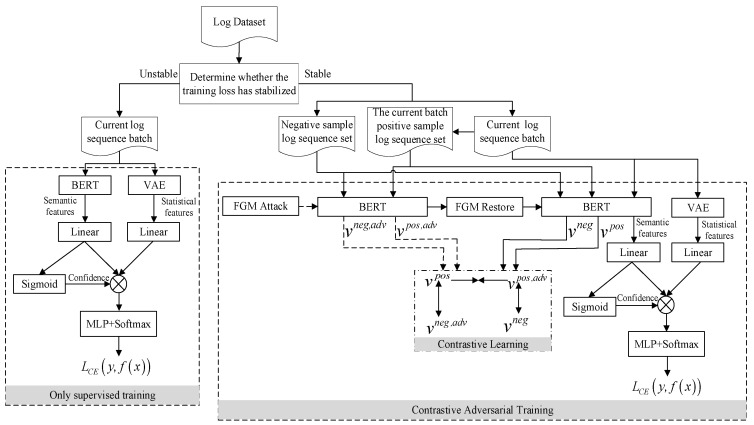
The specific process of contrastive adversarial training.

The specific process of the model using contrastive adversarial training in this paper is shown in Figure 6. It can be seen from the figure that before the training starts, m abnormal log sequences are randomly selected from the training set to form a negative sample set, and the value range of m is a positive integer less than the total number of negative samples in the training set. At the beginning of training, the log sequences in the training set and the corresponding labels are used to supervise the training of the model. This is because if the model used contrastive adversarial training at the beginning, it will cause too much noise, making the model difficult to converge. Suppose a batch log sequence training set is ${\left\{x_{i},y_{i}\right\}}_{i=1}^{N}$, where N is the size of a batch, $x_{i}$ is the *i*-th log sequence in the training set, $y_{i}$ is the corresponding real label, and the training loss function is Formula (6).

(6) $$L_{CE}=-\frac{1}{N}\sum_{i=1}^{N}\left(y_{i}\cdot \log\left(f_{\alpha }\left(x_{i}\right)\right)+\left(1-y_{i}\right)\cdot \log\left(1-f_{\alpha }\left(x_{i}\right)\right)\right)$$

where f is the forward function of the model, α is the model parameter, and $f_{\alpha }\left(x_{i}\right)$ is the prediction result of the model. In the training phase, the loss function is minimized by adjusting the model parameters. When the training loss of the supervised task of the model tends to be stable, contrastive adversarial training is started. This paper uses the FGM algorithm to perturb the embedding layer of the BERT. The idea of the FGM algorithm is to perturb the trainable parameters in the model according to the gradient generated during training to achieve the purpose of combating training. The perturbation size imposed by the FGM algorithm on the trainable parameters is shown in the Formula (7).

(7) $$r_{adv}=\varepsilon \cdot g/{\left\|g\right\|}_{2}$$

Among them, ε is a hyperparameter, and ${\left\|g\right\|}_{2}$ represents the second norm of the gradient. The normal log sequence in the current batch is selected to construct a positive sample set, and after obtaining the semantic features of the disturbed positive and negative sample set, restore the disturbance of the embedding layer of BERT. Then, while continuing to maintain the original supervised training task, contrastive learning is used to increase the similarity between the semantic features generated by the normal log sequence when the embedding layer of BERT is undisturbed and disturbed, and the similarity between the semantic features generated by the set of the positive and negative samples is reduced. Suppose the semantic vector generated by a batch log sequence is ${\left\{v_{i}\right\}}_{i=1}^{N}$, where N is the size of a batch, and the semantic vector generated by all normal log sequences in a batch is ${\left\{v_{i}^{pos}\right\}}_{i=1}^{n}$, where n is the number of normal log sequences, and $v_{i}^{pos}$ represents the vector generated by the *i*-th normal log sequence, and the semantic vector generated by the disturbed normal log sequence is ${\left\{v_{i}^{pos,adv}\right\}}_{i=1}^{n}$,where $v_{i}^{pos,adv}$ represents the vector generated by the *i*-th normal log sequence after the embedding layer is disturbed. The semantic vector generated by the negative sample log sequence set is ${\left\{v_{i}^{neg}\right\}}_{i=1}^{m}$, where m is the number of abnormal log sequences in the negative sample log sequence set, and $v_{i}^{neg}$ represents the vector generated by the *i*-th abnormal log sequence, and the semantic vector generated by the disturbed negative sample log sequence set is ${\left\{v_{i}^{neg,adv}\right\}}_{i=1}^{m}$, where $v_{i}^{pos,adv}$ represents the vector generated by the *i*-th abnormal log sequence after the embedding layer is disturbed. The optimization problem of contrastive learning is defined as Formula (8).

(8) $$L_{contra}=L_{contra}^{orig}+L_{contra}^{adv}$$

(9) $$L_{contra}^{orig}=-\frac{1}{n}\sum_{i=1}^{n}\log\frac{e^{sim\left(v_{i}^{pos},v_{i}^{pos,adv}\right)/\tau }}{e^{sim\left(v_{i}^{pos},v_{i}^{pos,adv}\right)/\tau }+\sum_{j=1}^{m}e^{sim\left(v_{i}^{pos},v_{j}^{neg,adv}\right)/\tau }}$$

(10) $$L_{contra}^{adv}=-\frac{1}{n}\sum_{i=1}^{n}\log\frac{e^{sim\left(v_{i}^{pos,adv},v_{i}^{pos}\right)/\tau }}{e^{sim\left(v_{i}^{pos,adv},v_{i}^{pos}\right)/\tau }+\sum_{j=1}^{m}e^{sim\left(v_{i}^{pos,adv},v_{j}^{neg}\right)/\tau }}$$

Among them, sim is the cosine similarity calculation, τ is the hyperparameter temperature coefficient, and $L_{contra}^{orig}$ is the contrastive loss function based on the semantic vector of the normal log sequence generated when the embedding layer is not disturbed. Its purpose is to force the semantic vector of the normal log sequence generated after the embedding layer is disturbed to be close to the semantic vector of the normal log sequence generated by the original embedding layer. At the same time, make the semantic vector of the abnormal log sequence generated after the embedding layer disturbed and the semantic vector of the normal log sequence generated by the original embedding layer distant. $L_{contra}^{adv}$ refers to the contrastive loss function based on the semantic vector of the normal log sequence generated after the embedding layer is disturbed. Its purpose is to force the semantic vector of the normal log sequence generated by the original embedding layer to be close to the semantic vector of the normal log sequence generated after the embedding layer is disturbed, and at the same time make the semantic vector of the abnormal log sequence generated by the original embedding layer and the embedding layer the semantic vector of the normal log sequence generated after the embedding layer is disturbed is distant. $L_{contra}$ represents the sum of $L_{contra}^{orig}$ and $L_{contra}^{adv}$. In the process of contrastive learning, the original supervised training of the model is also maintained, so the final optimization problem becomes Formula (11). The formula represents minimizing the sum of the cross-entropy loss function $L_{CE}$ of the original anomaly detection supervised task and the contrastive loss function $L_{contra}$ used to adjust the similarity between the semantic vector of the normal and abnormal log sequences by training the trainable parameter α of the model.

(11) $$\underset{\alpha }{\min}\left(L_{CE}+L_{contra}\right)$$

The robustness of log anomaly detection is largely reflected in the ability to more accurately understand the original semantic features expressed by the log sequence that has the updated log, thereby reducing the impact on the accuracy of anomaly detection. This paper uses contrastive learning to reduce the distance between the semantic features of the log sequence generated before and after the BERT disturbance, so that even if the input log sequence is subjected to an adversarial attack, the semantic features generated by it will not be significantly affected, so as to prevent adversarial attacks from leading the model to incorrectly understand the input log sequence. The robust BERT model trained in this way can still correctly understand the meaning of the log sequence when facing the log sequence that has the updated log, thereby improving the robustness of log anomaly detection.

## 4. Experimental Evaluation

In this section, the method of this paper is evaluated by studying the following aspects:

(1) The impact of the confidence threshold on the accuracy of anomaly detection.
(2) The effectiveness of the anomaly detection method in this paper.
(3) The robustness of the anomaly detection method in this paper.

### 4.1. Dataset and Experimental Environment

Dataset: This paper selects the real-world log data sets HDFS and BGL provided by LogHub [29] for experiments. The HDFS data set is collected by LogHub from the 203 node clusters of the Amazon EC2 platform. It is a common benchmark data for anomaly detection based on logs [30,31]. It contains a total of 11,175,629 original log messages, and 575061 sessions are assigned corresponding labels to indicate their normal and abnormal status. The BGL data set was collected by LogHub from the BlueGene/L supercomputer system of Lawrence Livermore National Laboratory (LLNL) [32] in Livermore, California. It contains a total of 4,747,963 original log messages, and each log has been marked as an alarm or non-alarm message. In the next experiment, for all data sets, 5000 normal log sequences and 5000 abnormal log sequences are selected from top to bottom based on the timestamp information of the logs. The first 80% are used as training data, and the remaining 20% are used as test data.

Experimental environment: The experiments in this paper are all carried out on the NVIDIA TESLA V100 32G GPU server. The Python 3.7 environment is used to build the model based on Pytorch, the Adam optimizer [33] is used to train the anomaly detection model, and the cross-entropy function is used as the loss function during training.

### 4.2. Baseline Methods and Indicators Evaluation

Baseline methods: This paper chooses SVM, DeepLog and LogRoust methods as the baseline method for comparison experiments.

Evaluation indicators: Anomaly detection is a binary classification problem. This paper uses widely used indicators, namely accuracy, recall, and F1-score to evaluate the accuracy of anomaly detection in this paper and various benchmark methods.

Accuracy: the percentage of log sequences that are truly abnormal in all log sequences judged to be abnormal by the model, as shown in Formula (12).

(12) $$\mathrm{Precision}=\frac{\mathrm{TP}}{\mathrm{TP}+\mathrm{FP}}$$

Recall: the percentage of all abnormal log sequences correctly identified as abnormal log sequences by the model, as shown in Formula (13).

(13) $$\mathrm{Recall}=\frac{\mathrm{TP}}{\mathrm{TP}+\mathrm{FN}}$$

F1 score: the harmonic average of precision rate and recall, as shown in Formula (14).

(14) $$F1\text{-}\mathrm{score}=\frac{2*\mathrm{Precision}*\mathrm{Recall}}{\mathrm{Precision}+\mathrm{Recall}}$$

Among them, TP is the number of abnormal log sequences correctly detected by the model. FP is the number of abnormal normal log sequences that the model incorrectly identified. FN is the number of abnormal log sequences that not detected by the model.

### 4.3. Experimental Parameter Settings

For the HDFS data set, the log sequence is obtained according to Block_id, and the label corresponding to the Block_id is used to determine whether the log sequence is abnormal. For the BGL data set, the log sequence is obtained by a sliding window with a size of 20. If there is an abnormal log in the log sequence, the entire log sequence is judged to be abnormal. For those methods that cannot cope with the updated log, this paper will correspond those new logs to a unified new dimension or new template to solve the situation that cannot cope with the log update, so as to facilitate the robustness comparison between methods. The size of the hyperparameter in the FGM algorithm is set to 1. The size of the negative sample log sequence collection is set to 32. The size of the temperature hyperparameter used to calculate the contrastive loss function is set to 0.05.

### 4.4. Test of the Influence of the Confidence Threshold on the Accuracy of Anomaly Detection

This paper conducts experiments on the hyperparameter η that controls the size of the confidence threshold to find its optimal value for the anomaly detection method in this paper. Specifically, when η is equal to 0, the semantic feature will discard all statistical features, and when η is equal to 0.5, the semantic feature will accept all statistical features. This paper uses five-fold cross validation to determine the hyperparameter η. Specifically, this paper divides the data set into five parts, and takes turns using four of them as training data and one as test data for experimentation. Each test will get the corresponding accuracy rate. The average of the accuracy of the 5 results is used as the final result of the accuracy of the algorithm. The experimental results of cross validation on HDFS and BGL datasets are shown in Table 2 and Table 3. It can be seen from the table that the value of η is different for different data sets. For the HDFS data set, when η is equal to 0.3, the anomaly detection accuracy rate is the highest, and for the BGL data set, when η is 0.2, the anomaly detection accuracy rate is the highest. For subsequent experiments, this paper will use η equal to 0.3 and 0.2 on the HDFS and BGL datasets to conduct experiments.

### 4.5. Test of Effectiveness

**Figure 7:**
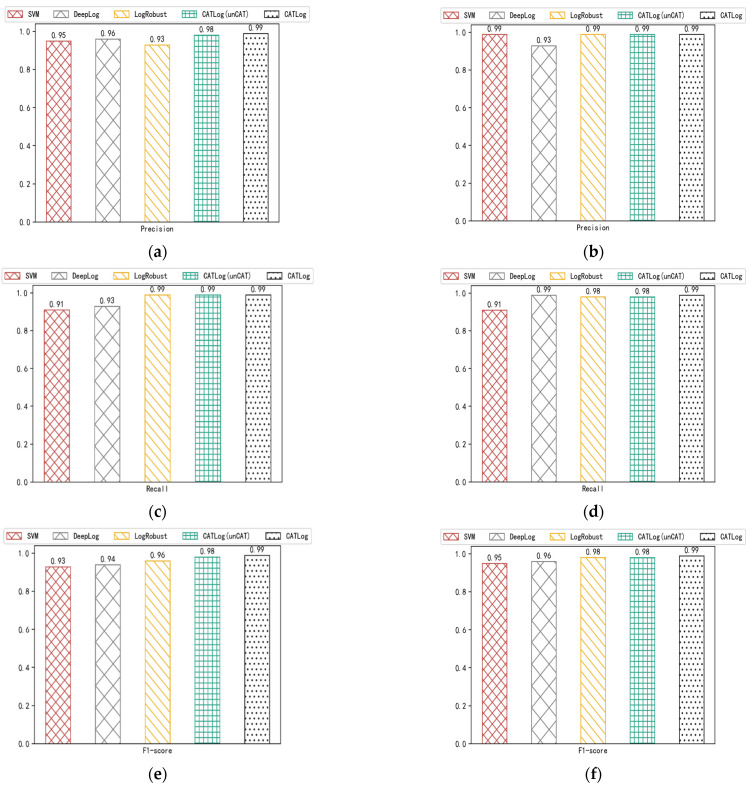
(**a**) Comparison of accuracy of different methods on HDFS; (**b**) Comparison of accuracy of different methods on BGL; (**c**) Comparison of recall of different methods on HDFS; (**d**) Comparison of recall of different methods on BGL; (**e**) F1-score comparison of different methods on HDFS; (**f**) F1-score comparison of different methods on BGL.

In order to prove the effectiveness of the CATLog proposed in this paper for anomaly detection, we compare the CATLog with a CATLog that has not undergone contrastive adversarial training and dual feature extraction and other baseline methods. The SVM anomaly detection method converts the log sequence into a count vector by counting the occurrence frequency of various log levels in the log sequence, and then realizes the abnormal detection of the log sequence by dividing the count vector. DeepLog uses LSTM to learn the normal trajectory generated by the log in the log sequence to find those log sequences that deviate from the normal trajectory, and then realize anomaly detection. LogRobust uses natural language processing methods to convert log templates into semantic vectors and uses a Bi-LSTM with an attention mechanism to perform supervised training on log sequences to achieve anomaly detection. Figure 7 (ace is the HDFS data set, bdf is the BGL data set) shows the comparison results of CATLog with CATLog that has not undergone contrastive adversarial training and dual feature extraction and other baseline methods on HDFS and BGL data sets.

It can be seen from the figure that the various anomaly detection indicators of the CATLog method proposed in this paper are higher than other baseline methods, and by comparing with the CATLog that has not undergone contrastive adversarial training and dual feature extraction, it can be seen that the CATLog that has undergone contrastive adversarial training and dual feature extraction has higher anomaly detection accuracy. The effectiveness of this method in anomaly detection has been confirmed.

In order to illustrate the effectiveness of the method in this paper more specifically, we conduct a “Friedman test”. Specifically, first, this paper compares the CATLog with the other three baseline methods on the HDFS and BGL data sets, and then sorts them on each data set according to the F1-score and assigns ordinal values (1, 2, …). As shown in Table 4, the last row is the average ordinal values. Then, the “Friedman test” is used to judge whether the performance of these models is the same. Let $r_{i}$ be the average ordinal values of *i*-th models. And we assume a variable $r_{\chi^{2}}=\frac{k-1}{k}\cdot \frac{12N}{k^{2}-1}\sum_{i=1}^{k}(r_{i}-\frac{k+1}{2}{)}^{2}$, where k=5 is the number of models, N=2 is the number of datasets. And we obtain the $r_{\chi^{2}}=7.7$, when k and N are large, $r_{\chi^{2}}$ obey distribution $\chi^{2}$ distribution. Then we use the variable $r_{F}=\frac{(N-1)r_{\chi^{2}}}{N(k-1)-r_{\chi^{2}}}$, which follows the F distribution with degrees of freedom (k−1) and (k−1)(N−1). Then we obtain the $r_{F}=25.7$, by referring to the table of common critical values for F test, when significance level α=0.05, $r_{F}>6.388$. Therefore, the assumption that all models have the same performance is rejected, there are obvious differences between algorithm performance.

### 4.6. Test of Robustness

**Figure 8:**
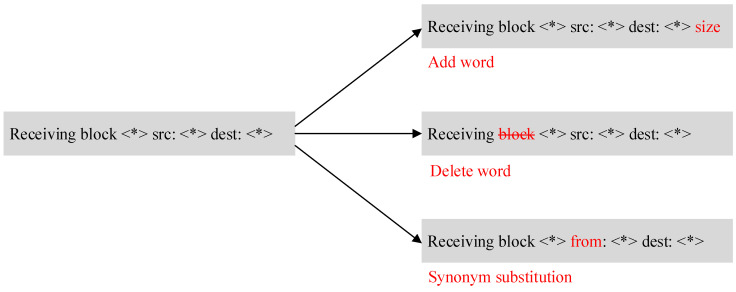
Update of log template.

With the upgrade of the system or service, some log template updates will occur to the log messages generated in the system. The irregular update of the log template will affect the accuracy of anomaly detection. Therefore, the robustness that can cope with the log template update becomes particularly important. In order to compare the robustness of CATLog with other baseline methods, this paper makes certain modifications to the original HDFS data set according to some log update rules. According to the related research on system log updates, it is found that the log update rules can be roughly divided into addition, deletion, and Synonymous substitution of log templates. The specific situation of the log update is shown in Figure 8.

**Figure 9:**
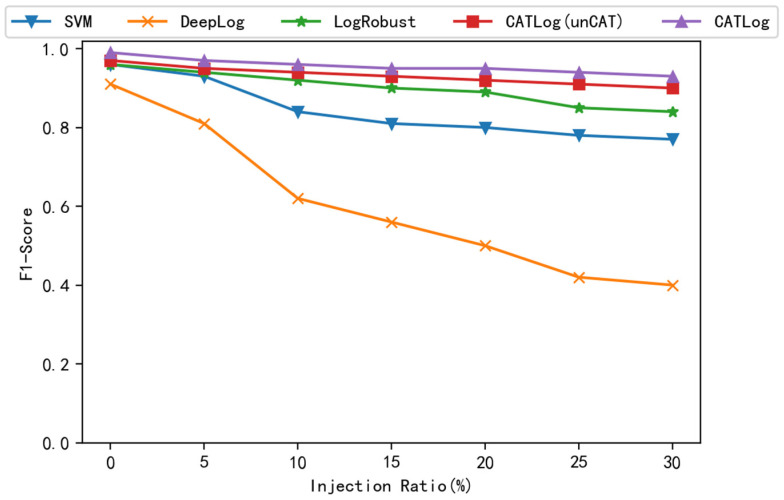
Comparison of robustness.

After a certain percentage of the original HDFS data set is updated, anomaly detection is performed again, and the comparison result of the F1 score is shown in Figure 9. As can be seen from the figure, when the update injection reaches 5%, the F1 scores of DeepLog begin to drop significantly. When the update injection reaches 10%, the F1 score of SVM and LogRobust also begins to drop significantly. Although the F1 score of the CATLog that has not undergone contrastive adversarial training and dual feature extraction is slightly higher than SVM and LogRobust, it is also lower than the CATLog that has undergone contrastive adversarial training and dual feature extraction. It can be concluded that the CATLog proposed in this paper has better robustness than the CATLog that has not undergone contrastive adversarial training and dual feature extraction and other baseline methods. The robustness of this method in anomaly detection has been confirmed.

## 5. Conclusions

This paper proposes a log sequence anomaly detection method CATLog based on contrastive adversarial training and dual feature extraction. The CATLog extracts the semantic features and statistical features in the log template. The core words in the abnormal log usually do not appear in the normal log, or appear in the normal log with a low probability, so the statistical features can help anomaly detection proceed more smoothly. CATLog uses contrastive learning to reduce the similarity between normal and abnormal log sequences, so that the model can better distinguish between normal and abnormal sequences. This can be well proven in the actual training process. This paper found that the classification loss of the CATLog is lower than that of the CATLog that has not undergone contrastive adversarial training when the accuracy of CATLog is trained to be the same as the CATLog that has not undergone contrastive adversarial training. CATLog uses contrastive adversarial training to conduct special training on the robustness of log anomaly detection, so that log entries can still guarantee the accuracy of anomaly detection after a certain update or some noise interference. This paper conducts comparative experiments on the real-world data set and the data set with updated part of the log template to evaluate the effectiveness and robustness of the anomaly detection method in this paper. The results show that this method is better than other methods. This paper analyzes the failure cases during the experiment and finds that the samples that fail to identify abnormalities are mostly caused by the scarcity of the samples during model training. In the future, this paper plans to collect more data sets to evaluate this method and solves the problem of a decrease in the accuracy of anomaly detection caused by the scarcity of samples.

## Figures and Tables

**Table 1:** Method comparison table.

Comparison Item Method	Input Value	Model or Algorithm	Strategy to Deal with Unseen Logs
SVM [5]	event count vector	construct a hyperplane	unable to deal with unseen logs
IM [2]	event count vector	singular value decomposition, brute force search algorithm	unable to deal with unseen logs
PCA [3]	event count vector	construct normal and abnormal subspaces	unable to deal with unseen logs
DeepLog [7]	logkey, parameter value	LSTM	unable to deal with unseen logs
CNN [10]	logkey	CNN	unable to deal with unseen logs
LogRobust [14]	semantic vector	Bi-LSTM with Attention	semantic vector conversion, attention mechanism
CATLog	semantic vector, statistical vector	BERT, VAE	semantic vector conversion, contrastive adversarial training

**Table 2:** The impact of the size of η on the accuracy of anomaly detection (HDFS dataset).

	*η* Value Size	0		0.1		0.2		0.3		0.4		0.5	
Round													
First round		0.985	0.985	0.985	0.985	0.986	0.986	0.988	0.988	0.987	0.987	0.987	0.987
Second round		0.984		0.986		0.987		0.989		0.988		0.987	
Third round		0.984		0.986		0.987		0.988		0.987		0.988	
Fourth round		0.986		0.985		0.986		0.987		0.987		0.987	
Fifth round		0.985		0.985		0.985		0.988		0.987		0.988	

**Table 3:** The impact of the size of η on the accuracy of anomaly detection (BGL dataset).

	*η* Value Size	0		0.1		0.2		0.3		0.4		0.5	
Round													
First round		0.988	0.987	0.989	0.988	0.990	0.991	0.989	0.990	0.987	0.988	0.987	0.989
Second round		0.987		0.988		0.991		0.990		0.989		0.988	
Third round		0.987		0.987		0.992		0.990		0.989		0.990	
Fourth round		0.987		0.988		0.990		0.989		0.988		0.990	
Fifth round		0.988		0.988		0.990		0.991		0.988		0.989	

**Table 4:** The ordinal values of different models.

Datasets	SVM	DeepLog	LogRobust	CATLog(unCAT)	CATLog
HDFS	5	4	3	2	1
BGL	5	4	2.5	2.5	1
The average ordinal values	5	4	2.75	2.25	1

## Data Availability

All the data used in the experiments can be downloaded from the following links: https://github.com/logpai/loghub, (accessed on 19 November 2021).
